# Effects of different plyometric training frequencies on physical performance in youth male volleyball players: a randomized trial

**DOI:** 10.3389/fphys.2023.1270512

**Published:** 2023-11-23

**Authors:** Jordan Hernandez-Martinez, Eduardo Guzman-Muñoz, Rodrigo Ramirez-Campillo, Tomas Herrera-Valenzuela, Braulio Henrique Magnani Branco, Sergio Avila-Valencia, Juan Luis Carter-Beltran, Pablo Aravena-Sagardia, Jorge Méndez-Cornejo, Pablo Valdés-Badilla

**Affiliations:** ^1^ Programa de Investigación en Deporte, Sociedad y Buen Vivir, Universidad de los Lagos, Osorno, Chile; ^2^ Department of Physical Activity Sciences, Universidad de Los Lagos, Osorno, Chile; ^3^ School of Kinesiology, Faculty of Health, Universidad Santo Tomás, Talca, Chile; ^4^ School of Kinesiology, Faculty of Health Sciences, Universidad Autónoma de Chile, Talca, Chile; ^5^ Exercise and Rehabilitation Sciences Institute, School of Physical Therapy, Faculty of Rehabilitation Sciences, Universidad Andres Bello, Santiago, Chile; ^6^ Department of Physical Activity, Sports and Health Sciences, Faculty of Medical Sciences, Universidad de Santiago de Chile (USACH), Santiago, Chile; ^7^ Postgraduate Program in Health Promotion, Cesumar University, Maringá, Paraná, Brazil; ^8^ Pedagogía en Educación Física, Facultad de Educación, Universidad Autónoma de Chile, Temuco, Chile; ^9^ Department of Physical Activity Sciences, Faculty of Education Sciences, Universidad Católica del Maule, Talca, Chile; ^10^ Sports Coach Career, School of Education, Universidad Viña del Mar, Viña del Mar, Chile

**Keywords:** muscle power, strength, plyometric exercise, sports, young, physical fitness

## Abstract

This study aimed to analyze the effect of plyometric training (PT) at different frequencies on jump performance, running sprint speed, and service speed in youth male volleyball players. The participants were randomly assigned to one PT session per week (Experimental Group 1, EG1, *n =* 15), two PT sessions per week (Experimental Group 2, EG2, *n =* 14), and a control group (CG, *n =* 13). The total weekly jumping ranged between 98 and 196 jumps (equalized between, EG1 and, EG2). The assessments performed were squat jump (SJ), countermovement jump (CMJ), CMJ-arms, drop jump (DJ), 5-m sprint, 10-m sprint, and service speed. The intragroup comparisons showed that, EG1 significantly (*p* < 0.001) improved SJ (*Δ* = 12.74%; *d* = 1.30), CMJ (*Δ* = 11.94%; *d* = 1.71), CMJ-arms (*Δ* = 12.02%; *d* = 1.47), DJ (*Δ* = 10.93%; *d* = 1.30), 5-m sprint (*Δ* = −4.61%; *d* = 0.29), 10-m sprint (*Δ* = −3.95%; *d* = 0.40) and service speed (*Δ* = 8.17%; *d* = 1.53). Similarly, EG2 significantly (*p*˂ 0.001) improved SJ (*Δ* = 11.52%; *d* = 1.25), CMJ (*Δ* = 11.29%; *d* = 1.38), CMJ-arms (*Δ* = 11.42%; *d* = 1.26), DJ (*Δ* = 13.90%; *d* = 2.17), 5-m sprint (*Δ* = −3.85%; *d* = 0.25), 10-m sprint (*Δ* = −2.73%; *d* = 0.25) and service speed (*Δ* = 6.77%; *d* = 1.44). The CG significantly (*p* < 0.05) improved SJ (*Δ* = 2.68; *d* = 0.28), CMJ-arms (*Δ* = 2.30; *d* = 0.35), 5-m sprint (*Δ* = −1.27; *d* = 0.10) and service speed (*Δ* = 1.42; *d* = 0.30). Intergroup comparisons revealed significantly greater improvements in all variables (*p* < 0.001) in, EG1 and, EG2 concerning to CG. However, no significant differences were found between, EG1 and, EG2. A moderate weekly PT volume, distributed in one or two sessions per week, seems equally effective.

## 1 Introduction

Volleyball is a sport of multidirectional movements that requires between 250 and 300 explosive actions, which occur repetitively during a match (Tramel et al., 2019). Among the most recurrent technical actions during the match are serving, setting, attacking, blocking (Pawlik et al., 2020), jumping, and ball spiking (Hernández Martínez, 2022). During a match, between 77% and 90% of jumps are executed, where outside attackers and middle blockers execute between 12 and 23 jumps per set (Lima et al., 2019), while ball spikes can reach >40,000 per year in elite players (Sarvestan et al., 2020). Therefore, it is important to implement training programs that enhance physical performance, focusing primarily on high-intensity actions such as jumping and serving (Oliveira et al., 2020; Ramirez-Campillo et al., 2020). Therefore, it is important to monitor the physical performance of volleyball players during competition (Sousa et al., 2023). This is achieved through the measurement and permanent monitoring of athlete’s performance and physical fitness (Sousa et al., 2023).

Plyometric training (PT) effectively improves vertical jump height and ball spiking in volleyball players (Silva et al., 2019; Ramirez-Campillo et al., 2021). In a study by Idrizovic et al. (2018) in youth female volleyball players (mean aged of 16.6 years), significant improvements in the countermovement jump (CMJ) of 16.9% (*p* < 0.05; *η* ^2^ = 0.29; *large effect*) were detected in favor of intervened with PT compared to a control group that performed a traditional training program. While Mroczek et al. (2019) detected significant improvements in the squat jump (SJ) test of 2.9% (*p* = 0.03; *moderate effect*) and 4.2% in CMJ (*p* = 0.00; *large effect*) following a 6-week PT intervention concerning the control group (no PT) in male high school volleyball players. In the study by Mackala et al. (2020), a PT intervention was carried out for 4 weeks in female high school volleyball players showing significant improvements in the drop jump (DJ) test of 7% (*p* = 0.00; *small effect*) compared to the control group. Similarly, an upper limbs-specific PT intervention performed for 8 weeks in adult volleyball players showed significant improvements (*p* < 0.001) in ball spiking by 3.8% compared to the control group (Valades Cerrato et al., 2018).

While PT at different periodizations (between 4 and 12 weeks) has been shown to positively affect physical performance in volleyball players (Idrizovic et al., 2018; Valades Cerrato et al., 2018), PT at different training frequencies (≤2 vs. >2 sessions per week) has been reported to lead to a significant improvement in vertical jump height in volleyball players (Ramirez-Campillo et al., 2021). In a systematic review by Silva et al. (2019) in volleyball players, they detected improvements located between 16.9% and 27.6% in CMJ and 5.2%–7.6% in 20-m sprint using interventions between 6 and 12 weeks of PT with a frequency of 2 and 3 sessions per week. In the study by Gjinovci et al. (2017), improvements of 8% in 20-m sprint and 27% in CMJ were reported by PT in a 12-week intervention with a frequency of 2 sessions per week. While Idrizovic et al. (2018), detected significant improvements by 16.9% in CMJ through a 12-week PT intervention with a frequency of 1 session per week in youth volleyball players. In contrast, Ramirez-Campillo et al. (2018), in a randomized controlled trial, compared different PT frequencies (1 session vs. 2 sessions per week) in amateur soccer players, reporting significant improvements in CMJ by 10.5% vs. 9.8% in DJ by 13.6% vs. 12.9% and in 15-m sprint by 8.2% vs. 9.6% compared to a control group. However, no significant differences were found when comparing PT groups. In another study conducted by Bouguezzi et al. (2020) in prepubertal soccer players comparing different PT frequencies (1 session vs. 2 sessions per week) without a control group, improvements were detected in both groups SJ 25.82% vs. 27.62%, in CMJ 23.41% vs. 20.04%, in 5-m sprint by −2.13% vs. −4.77% and in 10-m sprint by −1.10% vs. −2.13%. However, no significant differences were found when comparing PT groups.

Considering that PT at different frequencies both 1 and 2 sessions lead to significant improvements in physical performance in volleyball players (Gjinovci et al., 2017; Idrizovic et al., 2018; Ramirez-Campillo et al., 2020), it is necessary to identify the optimal dosage of jumps according to age, maturity and competitive level of the athletes to reduce the risk of injury (Ramirez-Campillo et al., 2023). To the best of our knowledge, only the effect of PT at different frequencies has been analyzed in a randomized controlled trial in amateur soccer players (Ramirez-Campillo et al., 2018). Therefore, the present study aimed to analyze the effect of PT at different frequencies on jump performance (SJ, CMJ, CMJ-arms, and DJ), running sprint speed (5-m sprint and 10-m sprint), and service speed in youth male volleyball players.

## 2 Methods

### 2.1 Study design

A single-blind, randomized, controlled trial of three parallel groups was conducted to compare the effects of 8 weeks of PT during the pre-season in youth male volleyball players. The experimental groups carried out PT sessions for 30 min, experimental group 1 (EG1) carried out one session per week of PT; experimental group 2 (EG2) carried out 2 training sessions per week, and the control group (CG) carried out a traditional volleyball training consisting of displacement exercises in different positions along with passing and spiking. Physical performance assessments were performed pre- and post-intervention consisting of SJ, CMJ, CMJ-arms, DJ, 5-m sprint, 10-m sprint, and service speed with both hands. One week before the start of the study, two familiarization sessions of 30 min each were conducted to explain and perform the test and training procedures to all participants to reduce possible learning effects.

### 2.2 Participants

**FIGURE 1:**
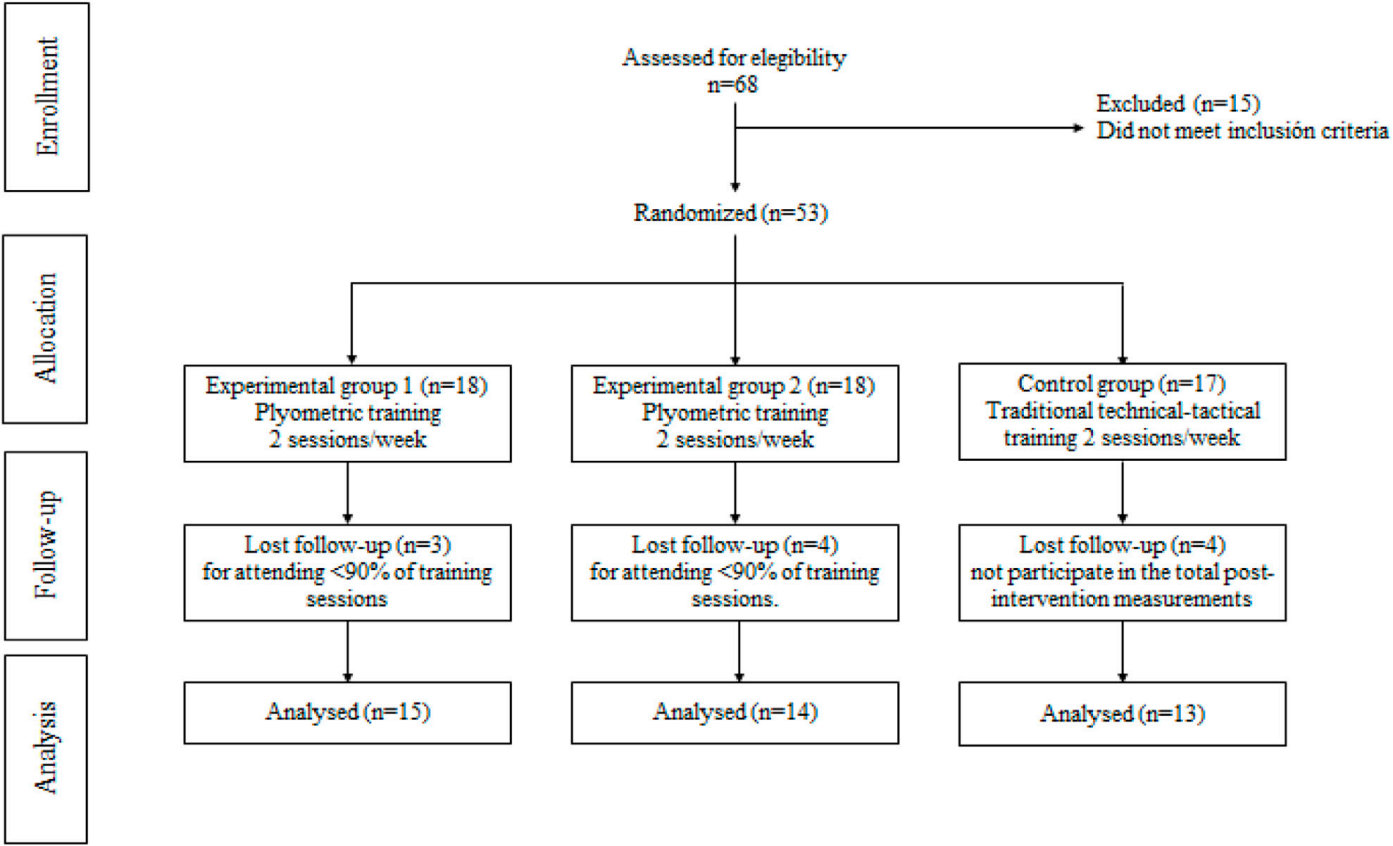
Flowchart of the recruitment process.

According to a previous study, the sample size calculation indicated that the ideal number of participants per group is 12 (Ramirez-Campillo et al., 2018). An alpha level of 0.05 was considered with a power of 80% with an effect size of d = 0.20. GPower software (version 3.1.9.6, Franz Faul, University Kiel, Germany) was used to calculate statistical power (Kang, 2021). The inclusion criteria were: *i*) have been practicing competitively for more than 6 months; *ii*) enrolled in federated clubs; *iii*) not having injuries that prevented them from performing the PT and physical performance assessments; *iv*) having the appropriate sports clothing to carry out the procedures; *v*) not being training in another club or national team attached to the existing one; *vi*) not being in competitions on the same days in which the PT were performed; *vii*) no systematic experience in PT during the last 6 months (*viii*) absence of any surgery of the lower and upper limbs in the last 2 years. Exclusion criteria were considered: *i*) those who presented cardiovascular or musculoskeletal pathologies that prevented them from performing the PT sessions; *ii*) not participating in all PT sessions. Sixty-eight youth male volleyball players from the Osorno, Chile, school league were recruited. Fifteen participants were excluded because they did not meet the inclusion criteria. Subsequently, 53 participants (aged = 14.7 ± 0.26 years, body mass = 58.1 ± 1.47 kg, bipedal height = 1.66 ± 3.66 m, body mass index [BMI] = 20.6 ± 1.15 kg/m^2^) were randomly assigned to: EG1 (*n =* 18), EG2 (*n =* 18), CG (*n =* 17). Eleven participants were excluded because: 4 did not participate in the total post-intervention measurements, and 7 did not complete ≥90% of the interventions. Therefore, the final sample included 15 participants in, EG1, 14 in, EG2, and 13 CG, who were considered for the subsequent analyses. No injuries were reported during the performance of the PT sessions and physical performance assessments. The sample selection process is presented in Figure 1 and the general characteristics of the sample in Table 1.

**TABLE 1:** Baseline characteristics of the sample.

	EG1 (*n =* 15)	EG2 (*n =* 14)	CG (*n =* 13)
Age (years)	14.6 ± 0.89	14.5 ± 0.57	15.0 ± 0.85
Body mass (kg)	57.4 ± 12.0	57.1 ± 6.87	59.8 ± 14.8
Height m)	1.69 ± 0.11	1.68 ± 0.07	1.62 ± 0.08
BMI (kg/m^2^)	19.7 ± 1.82	20.2 ± 1.82	21.9 ± 5.69
Experience (years)	4.65 ± 2.34	4.34 ± 2.12	4.78 ± 3.42
Training per week (days)	3	3	3
Training per week (min)	90	90	90
Competitive level	youth team	youth team	youth team

Data were presented as mean and (±) standard deviation; CG: control group; E. G1: Experimental Group 1; EG2: Experimental Group 2; kg: kilograms; m: meters; BMI: body mass index.

All participants and their legal guardians accepted the criteria for using and handling the data by providing assent and signed informed consent, authorizing the use of the information for scientific purposes. The research protocol was reviewed and approved by the Scientific Ethics Committee of the Universidad Autónoma de Chile (approval number: N° 126-18) and developed following the guidelines of the Helsinki Declaration statement for studies with human beings.

### 2.3 Anthropometric measurements

Bipedal height was measured using the Frankfort plane in a horizontal position, with a tape measure (Bodymeter 206, SECA, Germany; accuracy 0.1 cm) attached to the wall. Body mass was measured with an electronic scale (Omron HBF 514: accuracy 0.1 kg), while BMI was calculated by dividing body mass by bipedal height squared (kg/m^2^). All measurements were performed according to the recommendations of the International Society for the Advances in Kinanthropometry (ISAK) (Marfell-Jones et al., 2012).

### 2.4 Jump performance

All jumping tests were performed according to previous recommendations (Bosco et al., 1983). For the CMJ, volleyball players executed maximal effort jumps on an Ergojump^®^ Globus mobile contact platform (ErgoTest, Codogne, Italy) with arms over the iliac crests. Take-off and landing were standardized at the exact location, and players executed full knee and ankle extensions during the flight phase. The CMJ arms were performed similarly to the CMJ, except the upper limbs had freedom of action. For the SJ, the players stepped on the contact platform with arms over the iliac crest and a semi-flexed knee position at a 90° angle and the “stop” signal; the player maintained this posterior position, performing the maximum jump. The take-off and landing were standardized at the exact location, and the players executed full knee and ankle extensions during the flight phase. In the DJ test, participants were instructed to minimize ground contact time (<250 ms) after descending from a 20-cm box (Ramirez-Campillo et al., 2021). The best of three jumps (with a 1-min rest between each attempt) was recorded in CMJ, CMJ-arms, SJ, and DJ.

### 2.5 Running sprint speed

Sprint time was assessed to an accuracy of 0.01 s using Brower^®^ Timing System single-beam timing gates (Salt Lake City, Utah, United States). Participants started by positioning themselves behind the starting line. The sprint began when the participants started the event, automatically triggering the timing. Timing gates were placed at the start (0.3 m in front of the participant) at 5-m sprint and 10-m sprint. They were placed ∼0.7 m above the ground (approximately hip height). This system allows trunk movement to be captured trunk movement rather than the false triggering of a limb. Three sprints were performed, recording the best of the three sprints with 1 minute rest between each attempt (Sebastia-Amat et al., 2020).

### 2.6 Service speed

The youth volleyball players performed a service-like action to measure the ball’s maximum speed (km/h). The players used the tennis serve technique and were placed 2 m behind the end line of the court for the execution; the evaluator was located behind the end line of the opposing field, 20 m from the athlete, and pointed the radar gun for measurement to the participant. It was requested to serve with the maximum possible force to reach the opposite court’s interior. The serve was executed without jumps, given its influence on speed, and the ball could not touch the net. The players were instructed to serve inside the volleyball court, with the measurements of 9 m wide by 18 m long and a net height in the center of the field at 2.43 m. According to the International Volleyball Federation (FIV) regulations, a volleyball ball between 65 and 67 cm (Mikasa V200W) with an inflation pressure of 0.3–0.325 km/cm^2^ was used. Maximum speed was measured with a radar gun (Speed Gun SR3600; Sports Radar^®^, Homosassa, Florida, United States) with a pressure of 3%/1 Mile per hour (MPH) or 1 Kilometer per hour (Km/h) (Telles et al., 2021). Three attempts were carried out, recording the best of the three, with a minimum rest of 1 minute between each (Valades et al., 2016).

### 2.7 Training program

**FIGURE 2:**
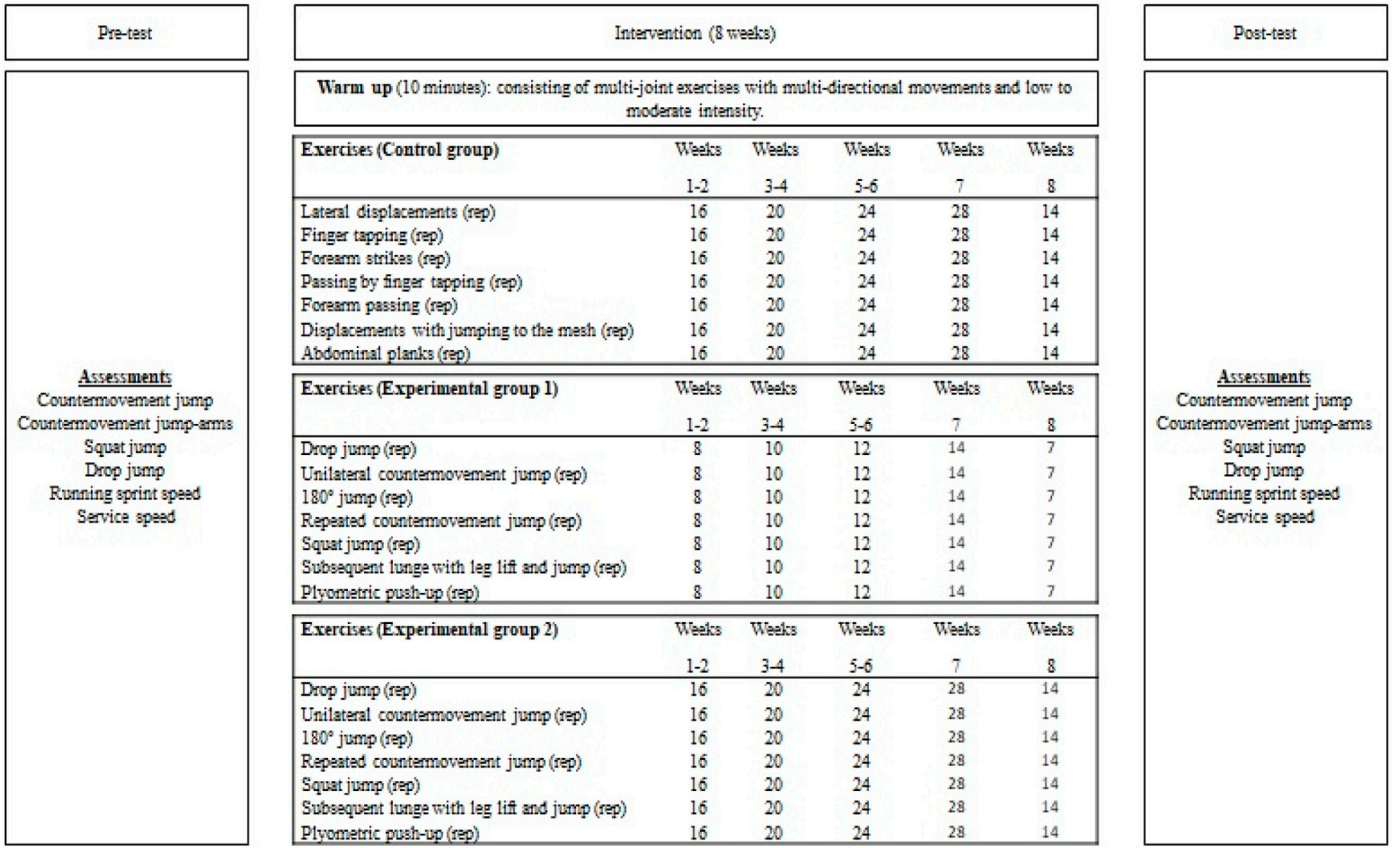
Assessments and training program.

A traditional volleyball training session consists of three parts: *i*) analytical: technical work, individual, doubles, finger, and forearm shots; *ii*) synthetic: physical-technical work, passing, attacking, and blocking; and *iii*) global: controlled games. The training program was performed during the pre-season. The exercises, sets, repetitions, and progressions per week are detailed in Figure 2. The CG continued their traditional volleyball training (i.e., mainly technical-tactical exercises, displacements in different directions, passes by finger and forearm strikes, ball spikes, and exercises to prevent injuries). The design of the PT intervention was based on the records in a previous modified study (Ramirez-Campillo et al., 2018). Unilateral, bilateral, cyclic (i.e., repeated), acyclic (i.e., non-repetitive), vertical, horizontal, and twisting jumping exercises were included, in addition to fast (<250 ms foot-to-ground contact time) and slow (≥250 ms ground contact time) muscle contractions of the stretch-shortening cycle, with a strong emphasis on PT landing and cushioning technique, using a medium hardness surface with PVC flooring (polyurethane) for the indoor gymnasiums. The PT was not added to traditional volleyball training but was performed immediately after the warm-up in replacement of some low to moderate-intensity volleyball technical-tactical exercises measured with the ten-point rating of perceived exertion (RPE) (Borg, 1982). Therefore, PT was conducted within the regular 90-min training period in which, EG1 conducted one PT session per week, and, EG2 conducted two PT sessions per week during the 8-week intervention period. The volume of total weekly jumping was equalized between, EG1 and, EG2, starting with 112 jumps between weeks 1–2, 140 jumps between weeks 3–4, 168 jumps between weeks 5–6, 196 jumps in week 7 and, 98 jumps for week 8. Each PT session included a set of 7 different jumping exercises, with 7–14 repetitions per set for, EG1 and 14 to 28 repetitions per set for, EG2. The jumping exercises were: *i*) DJ; *ii*) unilateral CMJ; *iii*) 180° jumps; *iv*) repeated CMJ; *v*) SJ; *vi*) reverse lunge to single leg skip jump; *vii*) plyometric push-up. During the DJ exercise, players were instructed to maximize the relationship between vertical height and ground contact time. It should be noted that players used individualized box heights; this was individualized by calculating the optimum reactive force index (i.e., from 5 to 35 cm) (Ramirez-Campillo et al., 2018).

The exercises were ordered and randomized each week, varying the training and avoiding possible monotony. The training volume was progressively increased from the first week to the seventh training week. During the eighth week, an adjustment strategy in terms of training volume was applied. During all training sessions, a researcher-participant ratio of 1:7 was applied, and special attention was paid to the technical execution of jumps. All PT sessions lasted between 12 and 25 min. The training sessions for, EG2 were twice as long as those for, EG1 due to the higher training volume per session. However, the total number of jumps performed during the entire training intervention (i.e., 8 weeks) was similar for both groups because the number of jumps performed during one session was equally distributed over two sessions. Both PT groups trained simultaneously (from 18:30 to 20:00 h), with the same rest intervals between exercise sets (i.e., 30–60 s). The intensities for both groups in the intervention sessions during the 8 weeks ranged from 3 to 6 RPE.

### 2.8 Statistical analysis

Data were presented as the mean and standard deviation, and statistical analyses were performed using the GraphPad Prism 8 (GraphPad Software, Inc, La Jolla, CA). The intraclass correlation coefficient (ICC) was calculated to determine the test-retest reliability of the physical performance assessments through a two-way mixed model consistency type. The Shapiro-Wilk normality test was used to determine the data distribution. The 3 × 2 mixed ANOVA model with repeated measures was performed to measure the group×time effect of all variables. When the group×time interaction was significant, Bonferroni *post hoc* test was performed to establish intragroup differences (pre vs. post) and intergroup differences (CG vs. EG1 vs. EG2). The partial eta squared (*η*_p_^2^) was calculated to determine the effect size of the group×time interaction, which was interpreted considering the ηp^2^ values of 0.01, 0.06, and 0.14, which correspond to an effect size small, medium, and large, respectively (Pallant, 2011). For multiple comparisons, the effect size was calculated using Cohen’s *d* (Cohen, 1992); considering a small (0.20–0.49), moderate (0.50–0.79), or large (≥0.80) effect, the formula used was d= (M1-M2)/SD (Rendón-Macías et al., 2021). Besides, the relative delta *Δ* also was calculated. A significance level of *p* < 0.05 was established.

## 3 Results

The reliability analysis of the physical performance assessments demonstrated large reliability values. The vertical jump height, as measured using the CMJ, showed a high reliability of 0.98. Similarly, CMJ-arms exhibited a reliability score of 0.92, while SJ and DJ boasted reliabilities of 0.95 and 0.91, respectively, reflecting a high level of consistency. Additionally, the data obtained for maximum velocity during 5-m and 10-m sprints exhibited high reliability, with an ICC of 0.95. Furthermore, the data collected for service speed demonstrated a high reliability of 0.93.

The group×time repeated measures ANOVA revealed a significant interaction for SJ (F_(2,38)_ = 30.53; *p* < 0.001; *η*_p_^2^ = 0.616; *large effect*), CMJ (F_(2,38)_ = 32.39; *p* < 0.001; *η*_p_^2^ = 0.630; *large effect*), CMJ-arms (F_(2,38)_ = 43.57; *p* < 0.001; *η*_p_^2^ = 0.696; *large effect*), DJ (F_(2,38)_ = 12.00; *p* < 0.001; *η*_p_^2^ = 0.387; *large effect*), 5-m sprint (F_(2,38)_ = 15.82; *p* < 0.001; *η*_p_^2^ = 0.454; *large effect*), 10-m sprint (F_(2,38)_ = 6.79; *p* < 0.003; *η*_p_^2^ = 0.263; *large effect*), and service speed (F_(2,38)_ = 50.98; *p* < 0.001; *η*_p_^2^ = 0.728; *large effect*).

Intragroup multiple comparisons showed that, EG1 improved performance in SJ (*p* < 0.001; *d* = 1.30; *large effect*), CMJ (*p* < 0.001; *d* = 1.71; *large effect*), CMJ-arms (*p* < 0.001; *d* = 1.47; *large effect*), DJ (*p* < 0.001; *d* = 1.30; *large effect*), 5-m sprint (*p* < 0.001; *d* = 0.29; *small effect*), 10-m sprint (*p* < 0.001; *d* = 0.40; *small effect*), and service speed (*p* < 0.001; *d* = 1.53; *large effect*). In the same way, EG2 showed significant improvement in SJ (*p* < 0.001; *d* = 1.25; *large effect*), CMJ (*p* < 0.001; *d* = 1.38; *large effect*), CMJ-arms (*p* < 0.001; *d* = 1.26; *large effect*), DJ (*p* < 0.001; *d* = 2.17; *large effect*), 5-m sprint (*p* < 0.001; *d* = 0.25; *small effect*), 10-m sprint (*p* < 0.001; *d* = 0.25; *small effect*), and service speed (*p* < 0.001; *d* = 1.44; *large effect*). In this intervention, the greatest changes in, EG2 were observed in SJ and DJ tests with a percentage change of *Δ* = 11.5% and *Δ* = 13.9%, respectively. On the other hand, in CG, significant improvements were observed in SJ (*p* = 0.040; *d* = 0.28; *small effect*), CMJ-arms (*p* = 0.031; *d* = 0.35; *small effect*), 5-m sprint (*p* = 0.015; *d* = 0.10; *small effect*), and service speed (*p* = 0.024; *d* = 0.30; *small effect*). The highest change percentages in CG were shown in SJ and DJ, with *Δ* = 2.7% and *Δ* = 3.8%, respectively. Table 2 presents intragroup multiple physical performance comparisons in youth male volleyball players.

**TABLE 2:** Intragroup multiple comparisons on physical performance in youth male volleyball players.

Assessments	Groups	PRE		Post		*p-*Value^#^	Change (%)	ES
		Mean	SD	Mean	SD			
Squat jump (cm)	CG (*n* = 12)	30.92	3.09	31.75	2.93	**0.040** ^*^	2.68	0.28
	EG1 (*n* = 15)	31.40	2.72	35.40	3.40	**<0.001** ^***^	12.74	1.30
	EG2 (*n* = 14)	31.00	2.63	34.57	3.06	**<0.001** ^***^	11.52	1.25
CMJ (cm)	CG (*n* = 12)	31.75	2.22	32.00	2.17	0.999	0.79	0.11
	EG1 (*n* = 15)	32.40	2.41	36.27	2.12	**<0.001** ^***^	11.94	1.71
	EG2 (*n* = 14)	31.00	2.63	34.50	2.44	**<0.001** ^***^	11.29	1.38
CMJ-arms (cm)	CG (*n* = 12)	32.67	2.35	33.42	1.98	**0.031** ^*^	2.30	0.35
	EG1 (*n* = 15)	33.27	2.60	37.27	2.84	**<0.001** ^***^	12.02	1.47
	EG2 (*n* = 14)	31.86	2.91	35.50	2.85	**<0.001** ^***^	11.42	1.26
Drop jump (cm)	CG (*n* = 12)	28.75	2.67	29.83	2.33	**0.098**	3.76	0.43
	EG1 (*n* = 15)	30.47	2.39	33.80	2.73	**<0.001** ^***^	10.93	1.30
	EG2 (*n* = 14)	30.79	2.05	35.07	1.90	**<0.001** ^***^	13.90	2.17
5-m sprint s)	CG (*n* = 12)	1.57	0.21	1.55	0.20	**0.015** ^*^	−1.27	0.10
	EG1 (*n* = 15)	1.52	0.22	1.45	0.26	**<0.001** ^***^	−4.61	0.29
	EG2 (*n* = 14)	1.56	0.20	1.50	0.28	**<0.001** ^***^	−3.85	0.25
10-m sprint s)	CG (*n* = 12)	2.58	0.27	2.56	0.26	**0.620**	−0.78	0.08
	EG1 (*n* = 15)	2.53	0.27	2.43	0.23	**<0.001** ^***^	−3.95	0.40
	EG2 (*n* = 14)	2.56	0.28	2.49	0.28	**<0.001** ^***^	−2.73	0.25
Service speed (km/h)	CG (*n* = 12)	59.08	2.81	59.92	2.81	**0.024** ^*^	1.42	0.30
	EG1 (*n* = 15)	57.87	3.07	62.60	3.11	**<0.001** ^***^	8.17	1.53
	EG2 (*n* = 14)	57.00	2.94	60.86	2.41	**<0.001** ^***^	6.77	1.44

Data were presented as mean and (±) standard deviation; *SD: standard deviation. CMJ: countermovement jump. ES: effect size*.

*p*< *0.05.*

*p*< *0.01.*

*p*< *0.001*.

*Bonferroni post hoc test*.

Values marked in bold show that there are statistically significant differences. *p* < 0.05*, *p* < 0.01**, *p* < 0.001***.

Intergroup multiple comparisons revealed significant differences in all the variables assessed in favor of, EG1 and, EG2 concerning CG (*p* < 0.001), with no differences between, EG1 and, EG2. Table 3 presents multiple intergroup comparisons on the effects of different PT frequencies on physical performance in youth male volleyball players.

**TABLE 3:** Intergroup multiple comparisons on the effects of different plyometric training frequencies on physical performance in youth male volleyball players.

Assessments	CG vs. EG1	CG vs. EG2	EG1 vs. EG2
Squat Jump (cm)	***p*< 0.001** ^***^	*p*= 0.001**	*p* = 0.999
	ES = 1.15	ES = 0.94	ES = 0.25
CMJ (cm)	*p*= 0.001**	***p*< 0.001** ^***^	*p* = 0.999
	ES = 1.99	ES = 1.08	ES = 0.47
CMJ-arms (cm)	***p*< 0.001** ^***^	***p*< 0.001** ^***^	*p* = 0.999
	ES = 1.57	ES = 0.84	ES = 0.62
Drop Jump (cm)	*p*= 0.001**	***p*< 0.001** ^***^	*p* = 0.999
	ES = 1.56	ES = 2.42	ES = 0.53
5-m sprint s)	***p*< 0.001** ^***^	***p*< 0.001** ^***^	*p* = 0.999
	ES = 0.43	ES = 0.20	ES = 0.18
10-m sprint s)	***p*< 0.001** ^***^	*p*= 0.001**	*p* = 0.999
	ES = 0.53	ES = 0.25	ES = 0.23
Service speed (km/h)	***p*< 0.001** ^***^	***p*< 0.001** ^***^	*p* = 0.999
	ES = 0.90	ES = 0.62	ES = 0.35

Bonferroni *post hoc* test.

*p*< *0.05.*

*p*< *0.01.*

*p*< *0.001. p*: *p*-value. *ES: effect size. CG: control group. E.G.,1: experimental group 1. E.G.,2: experimental group 2. CMJ: countermovement jump*.

Values marked in bold show that there are statistically significant differences. *p* < 0.05*, *p* < 0.01**, *p* < 0.001***.

## 4 Discussion

The present study aimed to analyze the effect of PT at different frequencies on jump performance (SJ, CMJ, CMJ-arms, DJ), running sprint speed (5-m sprint, 10-m sprint), and service speed in youth male volleyball players. Our main outcomes showed that both interventions with PT in one (EG1) and two sessions (EG2) weekly were equally effective in improving the components of physical performance in youth volleyball players under controlled training volume, these being statistically significant improvements in jump performance, running sprint speed, and service speed. Therefore, one weekly PT session has a similar effect to two weekly sessions during the pre-season (8 weeks) on physical performance in youth volleyball players.

In the present study, significant improvements in SJ and CMJ-arms in CG and, after PT intervention were detected in both, EG1and, EG2 in SJ, CMJ, CMJ-arms and DJ in youth male volleyball players. These findings confirm the results of previous systematic reviews indicating significantly greater responses in volleyball players who intervened with PT in SJ (*p* < 0.05; *d* = 0.56; *moderate effect*), CMJ (*p* < 0.05; *d* = 0.80; *large effect*), and DJ (*p* < 0.05; *d* = 0.81; *large effect*) relative to active control groups (Ramirez-Campillo et al., 2021). A study conducted by Ramirez-Campillo et al. (2018) compared 1 and 2 weekly sessions of a PT intervention and found that it significantly improved SJ, CMJ, and DJ compared to the active control group in youth amateur soccer players. These improvements reported in the present study in jump performance are important because, in volleyball, actions such as blocking and spiking during a match are executed by jumping, and 80% of the points in a match are obtained by performing these actions (Carvalho et al., 2020). Therefore, a higher vertical jump height is decisive for match performance (Carvalho et al., 2020). In the systematic review with meta-analysis performed by Ramirez-Campillo et al. (2020), it was observed that both a number of PT sessions ≤16 sessions vs. >16 sessions led to significant improvements (*p* < 0.05) in vertical jump height in volleyball players with a moderate effect size (ES = 0.730–0.916) with no significant differences between groups (*p* = 0.558). As well as the number of jumps per session, ∼42 jumps vs. ∼160 jumps per session both lead to a significant improvement (*p* < 0.05) in vertical jump height performance in volleyball players with a moderate effect size (ES = 0.761–0.785) with no significant differences between groups (*p* = 0.811). These improvements could be due to various neuromuscular adaptations, such as improvements in intermuscular coordination, increased activation rate of alpha motor neurons, improved mechanical characteristics of the muscle-tendon complex, improved muscle size, architecture, and/or single fiber mechanics (Ramirez-Campillo et al., 2021). However, it should be considered that a very high frequency of PT can lead to a higher risk of injury due to muscle fatigue in young volleyball players (Migliorini et al., 2019; Tsarbou et al., 2021; Pawlik and Mroczek, 2022). Therefore, one session a week can generate adaptations and improvements in vertical jump height in youth volleyball players.

Another reported improvement was in running sprint speed in CG, decreasing the time in 5-m sprint and, after PT intervention with both, EG1 and, EG2 in 5-m sprint and 10-m sprint. These results confirm what was reported in the meta-analysis by Ramirez-Campillo et al. (2021) in a 10-m sprint (ES = 0.70; 95%CI = 0.31 to 1.09; *p* < 0.001; I^2^ = 46.1%) in youth volleyball players. In amateur soccer players, significant improvements (*p* < 0.01) in the 15-m sprint through one and two PT sessions over 8 weeks compared to an active control group were reported (ES = 2.25 and ES = 2.67) with a magnitude of change of −8.3% and −9.5%. However, no significant differences were reported when comparing both PT groups. Volleyball is a team sport characterized by intermittent efforts with periods of short duration (i.e., 3–9 s), and high-intensity activities interspersed with relatively long (i.e., 10 s–20 s) recovery periods (Ramirez-Campillo et al., 2021). Short-distance sprints (5-m sprint and 10-m sprint) form a relatively larger portion (i.e., ∼30%) of the total movement distance in volleyball, particularly in linear sprints (Ramirez-Campillo et al., 2021). Likewise, a fast-running approach prior to jumping is also related to a better jump height (Ramirez-Campillo et al., 2021). Sprint performance requires explosive concentric force production and stretch-shortening cycle in the lower limb muscles and can benefit significantly from the ability of players to utilize and optimize the elastic and neural properties of the stretch-shortening cycle after PT (Silva et al., 2019). In a systematic review with meta-analysis Ramirez-Campillo et al. (2021) it has been shown that PT leads to significant improvements (*p* < 0.05) in linear sprinting of ≤10 m and >10 m in basketball players (≥16.3 years and <16.3 years) with training frequencies ≤2 sessions/week vs. >2 sessions/week. While in volleyball players (≥16 years and <16 years), the PT with frequencies of <8 weeks with <16 sessions/week vs. ≥ 8 weeks with ≥16 sessions/week led to significant improvements (*p* < 0.05) in a linear sprint with no significant differences between groups. Higher PT volume has been associated with increased injury risk (Brumitt et al., 2016; Brumitt et al., 2018). In this context, a previous study conducted among physically active participants revealed that varying jump volumes, including low (420 jumps), moderate (840 jumps), and high (1,680 jumps), resulted in comparable enhancements in linear sprint performance (Alfaro-Jiménez et al., 2018).

Also, in the present study, significant improvements in service speed in CG were reported in both, EG1 and, EG2. Similar to that reported by Valades Cerrato et al. (2018), showing significant improvements after PT in serving speed (*Δ* 3.8%) compared to the active control group in youth volleyball players. The explosive actions of the upper limbs, such as the power with which the ball is impacted in the service, determine success in the match (Valades Cerrato et al., 2018). The service is the first action through which a point can be scored, preceding all other scoring actions, such as the spike or the block (Quiroga et al., 2010). In a study conducted by Valades Cerrato et al. (2018), the actions that awarded the most points in the Sydney 2000 and Athens 2004 Olympic Games were the serve was between 4.4% and 8.1%, the spike of 76.8%–80%, and the block 14.5%–15.6%. The PT increases strength, speed, and muscle power in volleyball players’ lower and upper limbs (Hammami et al., 2020). These improvements could be due to various neuromuscular adaptions, such as improvements in intermuscular coordination, and transfers obtained through PT leading to higher service speed in volleyball players (Valades et al., 2016).

While PT can induce a wide range of adaptions, several specific features of PT must be considered to maximize its benefits (Ramirez-Campillo et al., 2018). In this study, the PT intervention included unilateral, bilateral, cyclic, acyclic, vertical, fast, and slow muscle contraction exercises of the stretch-shortening cycle, with movements similar to sports gestures, emphasizing landing technique and shock absorption, using soft and medium hardness training surfaces. Also, PT was performed immediately after warm-up, and participants used individualized box heights (i.e., 5–35 cm) during DJs, progressively increasing the training volume, and using adequate rest between sets and repetitions (Ramirez-Campillo et al., 2018). It should be noted that all the PT sessions were carried out on surfaces that the players usually use in training and competitions. Also, taking into account previous studies (Gjinovci et al., 2017; Idrizovic et al., 2018; Ramirez-Campillo et al., 2018; Ramirez-Campillo et al., 2021) in volleyball players. This is the first study to analyze the effect of a highly specialized PT program to maximize adaptations on lower and upper limbs performance variables in youth male volleyball players. However, considering that the effects of PT may vary according to sex, sport level, age, and years of experience (Ramirez-Campillo et al., 2021). In PT programs the distribution and quantity of jumps is important to achieve the best benefits, thus the scientific literature has proposed in male and female professional and elite volleyball players (aged between 14 and 21 years) to perform a duration ≤8 weeks, with a frequency ≤2 sessions/week with a total number ≤16 sessions performing a volume of jumps <2,000 (Ramirez-Campillo et al., 2020). This better stimulates fast twitch fibers, which would help improve performance, however, there is still no consensus on the number of weekly sessions for amateur or school league players, where maturity such as competition level may influence the duration and frequency of PT (Ramirez-Campillo et al., 2023).

Because of this, the limitations of the study are: *i*) not including measurements of neurophysiological mechanisms to determine muscle activation in response to the adaptations generated by PT; *ii*) not assessing the level of physical maturation of the participants; *iii*) the absence of female in the sample; *iv*) not assessment professional or elite athletes to determine whether the same responses are generated in response to PT. Among the main strengths of the study, we can mention: *i*) the randomization of the sample; *ii*) the execution of the same protocol in both PT groups; *iii*) the equal load between, EG1 and, EG2; *iv*) the surface on which the PT intervention was carried out.

Based on the results found, one PT session week in combination with traditional volleyball training seems to be sufficient to induce improvements in jump performance (SJ, CMJ, CMJ-arms, DJ), running sprint speed (5-m sprint, 10-m sprint) and a service speed of both lower and upper limbs in youth male volleyball players, with a moderate overall training volume, a different distribution (1 or 2 sessions) of a given weekly training volume does not produce statistically significant differences in training adaptations.

## 5 Conclusion

A single PT session per week for 8 weeks is adequate for achieving significant improvements in jump performance, running sprint speed, and service speed. These results are comparable to those obtained from two weekly PT sessions. Therefore, the distribution of one or two sessions per week does not yield statistically significant differences in physical performance among youth male volleyball players.

## Data Availability

The original contributions presented in the study are included in the article/Supplementary material, further inquiries can be directed to the corresponding author.

## References

[B1] Alfaro-Jiménez D. Salicetti-Fonseca A. Jiménez-Díaz J. (2018). Efecto del entrenamiento pliométrico en la fuerza explosiva en deportes colectivos: un metaanálisis. Pensar mov. Rev. Ciencias del Ejerc. Salud 16 (1), 27752. 10.15517/pensarmov.v16i1.27752

[B2] Borg G. A. (1982). Psychophysical bases of perceived exertion. Med. Sci. Sports Exerc 14 (5), 377–381. 10.1249/00005768-198205000-00012 7154893

[B3] Bosco C. Luhtanen P. Komi P. V. (1983). A simple method for measurement of mechanical power in jumping. Eur. J. Appl. Physiol. Occup. Physiol. 50 (2), 273–282. 10.1007/bf00422166 6681758

[B4] Bouguezzi R. Chaabene H. Negra Y. Ramirez-Campillo R. Jlalia Z. Mkaouer B. (2020). Effects of different plyometric training frequencies on measures of athletic performance in prepuberal male soccer players. J. Strength Cond. Res. 34 (6), 1609–1617. 10.1519/jsc.0000000000002486 32453304

[B5] Brumitt J. Heiderscheit B. C. Manske R. C. Niemuth P. Mattocks A. Rauh M. J. (2016). The lower-extremity functional test and lower-quadrant injury in NCAA division III athletes: a descriptive and epidemiologic report. J. Sport Rehabil. 25 (3), 219–226. 10.1123/jsr.2014-0316 25946403

[B6] Brumitt J. Wilson V. Ellis N. Petersen J. Zita C. J. Reyes J. (2018). Preseason lower extremity functional test scores are not associated with lower quadrant injury - a validation study with normative data on 395 division iii athletes. Int. J. Sports Phys. Ther. 13 (3), 410–421. 10.26603/ijspt20180410 30038827 PMC6044601

[B7] Carvalho A. Roriz P. Duarte D. (2020). Comparison of morphological profiles and performance variables between female volleyball players of the first and second division in Portugal. J. Hum. Kinet. 71, 109–117. 10.2478/hukin-2019-0076 32148576 PMC7052714

[B8] Cohen J. (1992). A power primer. Psychol. Bull. 112 (1), 155–159. 10.1037//0033-2909.112.1.155 19565683

[B9] Gjinovci B. Idrizovic K. Uljevic O. Sekulic D. (2017). Plyometric training improves sprinting, jumping and throwing capacities of high level female volleyball players better than skill-based conditioning. J. Sports Sci. Med. 16 (4), 527–535. 29238253 PMC5721183

[B10] Hammami M. Gaamouri N. Suzuki K. Shephard R. J. Chelly M. S. (2020). Effects of upper and lower limb plyometric training program on components of physical performance in young female handball players. Front. Physiol. 11, 1028. 10.3389/fphys.2020.01028 33013446 PMC7461999

[B11] Hernández Martínez J. Y. C. D. Cisterna D. A. (2022). Potencia muscular en relación a la composición corporal en jugadores de voleibol adolescentes según género. Ciencias Act. Física UCM 23 (1), 1–8. 10.29035/rcaf.23.1.10

[B12] Idrizovic K. Gjinovci B. Sekulic D. Uljevic O. João P. V. Spasic M. (2018). The effects of 3-month skill-based and plyometric conditioning on fitness parameters in junior female volleyball players. Pediatr. Exerc Sci. 30 (3), 353–363. 10.1123/pes.2017-0178 29478378

[B13] Kang H. (2021). Sample size determination and power analysis using the G*Power software. J. Educ. Eval. health Prof. 18, 17. 10.3352/jeehp.2021.18.17 34325496 PMC8441096

[B14] Lima R. F. Palao J. M. Clemente F. M. (2019). Jump performance during official matches in elite volleyball players: a pilot study. J. Hum. Kinet. 67, 259–269. 10.2478/hukin-2018-0080 31523323 PMC6714353

[B15] Mackala K. Synowka A. Corluka M. Vodicar J. (2020). Impact of plyometric training on the power of lower limbs in moderately advanced female volleyball players. Acta Kinesiol. 15, 5–12. 10.51371/issn.1840-2976.2021.15.S1.1

[B16] Marfell-Jones M. J. S. Stewart A. D. de Ridder J. H. (2012). International standards for anthropometric assessment. Wellington, New Zealand international society for the advancement of Kinanthropometry. Available at: http://hdl.handle.net/11072/1510.

[B17] Migliorini F. Rath B. Tingart M. Niewiera M. Colarossi G. Baroncini A. (2019). Injuries among volleyball players: a comprehensive survey of the literature. Sport Sci. Health 15 (2), 281–293. 10.1007/s11332-019-00549-x

[B18] Mroczek D. Mackala K. Chmura P. Superlak E. Konefal M. Seweryniak T. (2019). Effects of plyometrics training on muscle stiffness changes in male volleyball players. J. Strength Cond. Res. 33 (4), 910–921. 10.1519/jsc.0000000000003074 30789578

[B19] Oliveira L. D. S. Moura T. B. M. A. Rodacki A. L. F. Tilp M. Okazaki V. H. A. (2020). A systematic review of volleyball spike kinematics: implications for practice and research. Int. J. Sports Sci. Coach. 15 (2), 239–255. 10.1177/1747954119899881

[B20] Pallant J. (2011). SPSS survival manual: a step by step guide to data analysis using the SPSS program. 4th ed. Allen and Unwin, Berkshire. Available at: https://www.scirp.org/(S(i43dyn45teexjx455qlt3d2q))/reference/ReferencesPapers.aspx?ReferenceID=851962.

[B21] Pawlik D. Kawczyński A. Chmura J. Maćkała K. Kutrzyński M. Mroczek D. (2020). Jumping flying distance and jump performance of elite male volleyball players at FIVB volleyball men’s world championship. Appl. Sci. 10 (6), 2045. 10.3390/app10062045

[B22] Pawlik D. Mroczek D. (2022). Fatigue and training load factors in volleyball. Int. J. Environ. Res. Public Health 19 (18), 11149. 10.3390/ijerph191811149 36141425 PMC9517593

[B23] Quiroga M. E. García-Manso J. M. Rodríguez-Ruiz D. Sarmiento S. De Saa Y. Moreno M. P. (2010). Relation between in-game role and service characteristics in elite women's volleyball. J. Strength Cond. Res. 24 (9), 2316–2321. 10.1519/JSC.0b013e3181e3812e 20703161

[B24] Ramirez-Campillo R. Andrade D. C. Nikolaidis P. T. Moran J. Clemente F. M. Chaabene H. (2020). Effects of plyometric jump training on vertical jump height of volleyball players: a systematic review with meta-analysis of randomized-controlled trial. J. Sports Sci. Med. 19 (3), 489–499. 32874101 PMC7429440

[B25] Ramirez-Campillo R. García-de-Alcaraz A. Chaabene H. Moran J. Negra Y. Granacher U. (2021). Effects of plyometric jump training on physical fitness in amateur and professional volleyball: a meta-analysis. Front. Physiol. 12, 636140. 10.3389/fphys.2021.636140 33716784 PMC7952872

[B26] Ramirez-Campillo R. García-Pinillos F. García-Ramos A. Yanci J. Gentil P. Chaabene H. (2018). Effects of different plyometric training frequencies on components of physical fitness in amateur female soccer players. Front. Physiol. 9, 934. 10.3389/fphys.2018.00934 30065665 PMC6056896

[B27] Ramirez-Campillo R. Sortwell A. Moran J. Afonso J. Clemente F. M. Lloyd R. S. (2023). Plyometric-jump training effects on physical fitness and sport-specific performance according to maturity: a systematic review with meta-analysis. Sports Med. Open 9 (1), 23. 10.1186/s40798-023-00568-6 37036542 PMC10086091

[B28] Rendón-Macías M. E. Zarco-Villavicencio I. S. Villasís-Keever M. (2021). Métodos estadísticos para el análisis del tamaño del efecto. Rev. Alerg. Mex. 68 (2), 128–136. 10.29262/ram.v658i2.949 34525784

[B29] Sarvestan J. Svoboda Z. Linduška P. (2020). Kinematic differences between successful and faulty spikes in young volleyball players. J. Sports Sci. 38 (20), 2314–2320. 10.1080/02640414.2020.1782008 32965184

[B30] Sebastia-Amat S. Pueo B. Villalon-Gasch L. Jimenez-Olmedo J. M. (2020). Anthropometric profile and conditional factors of U21 Spanish elite beach volleyball players according to playing position (Perfil antropométrico y factores condicionales de los jugadores españoles élite sub-21 según la posición de juego). Retos 38 (0), 620–625. 10.47197/retos.v38i38.76766

[B31] Silva A. F. Clemente F. M. Lima R. Nikolaidis P. T. Rosemann T. Knechtle B. (2019). The effect of plyometric training in volleyball players: a systematic review. Int. J. Environ. Res. Public Health 16 (16), 2960. 10.3390/ijerph16162960 31426481 PMC6720263

[B32] Sousa A. C. Marques D. L. Marinho D. A. Neiva H. P. Marques M. C. (2023). Assessing and monitoring physical performance using wearable technologies in volleyball players: a systematic review. Appl. Sci. 13 (7), 4102. 10.3390/app13074102

[B33] Telles R. Cunha R. A. Yoshimura A. L. Pochini A. C. Ejnisman B. Soliaman R. R. (2021). Shoulder rotation range of motion and serve speed in adolescent male volleyball athletes: a cross-sectional study. Int. J. Sports Phys. Ther. 16 (2), 496–503. 10.26603/001c.21243 33842045 PMC8016436

[B34] Tramel W. Lockie R. G. Lindsay K. G. Dawes J. J. (2019). Associations between absolute and relative lower body strength to measures of power and change of direction speed in division II female volleyball players. Sports (Basel) 7 (7), 160. 10.3390/sports7070160 31266193 PMC6680823

[B35] Tsarbou C. Liveris N. I. Tsimeas P. D. Papageorgiou G. Xergia S. A. Tsiokanos A. (2021). The effect of fatigue on jump height and the risk of knee injury after a volleyball training game: a pilot study. J. Biomed. Hum. Kinet. 13 (1), 197–204. 10.2478/bhk-2021-0024

[B36] Valades D. Palao J. Aúnsolo Á. Ureña Espa A. (2016). Correlation between ball speed of the spike and the strength condition of a professional women’s volleyball team during the season. Kinesiology 48, 87–94. 10.26582/k.48.1.7

[B37] Valades Cerrato D. Palao J. M. Femia P. Urena A. (2018). Effect of eight weeks of upper-body plyometric training during the competitive season on professional female volleyball players. J. Sports Med. Phys. Fit. 58 (10), 1423–1431. 10.23736/s0022-4707.17.07527-2 28745472

